# Quantification of MUCIN 1, cell surface associated and MUCIN16, cell surface associated proteins in tears and conjunctival epithelial cells collected from postmenopausal women

**Published:** 2013-05-06

**Authors:** Sruthi Srinivasan, Miriam L. Heynen, Elizabeth Martell, Robert Ritter, Lyndon Jones, Michelle Senchyna

**Affiliations:** 1 Centre for Contact Lens Research, School of Optometry, University of Waterloo, Waterloo, Ontario, Canada; 2 Alcon Research Ltd, Fort Worth, TX

## Abstract

**Purpose:**

To quantify the expression of mucin 1, cell surface associated (MUC1) and mucin 16, cell surface associated (MUC16) proteins and messenger ribonucleic acid (mRNA) in a cohort of postmenopausal women (PMW), to explore the relationship between mucin expression, dry eye symptomology, and tear stability.

**Methods:**

Thirty-nine healthy PMW (>50 years of age) were enrolled in this study. No specific inclusion criteria were used to define dry eye; instead, a range of subjects were recruited based on responses to the Allergan Ocular Surface Disease Index (OSDI) questionnaire and tear stability measurements as assessed by non-invasive tear breakup time (NITBUT). Tears were collected from the inferior tear meniscus using a disposable glass capillary tube, and total RNA and total protein were isolated from conjunctival epithelial cells collected via impression cytology. Expression of membrane-bound and soluble MUC1 and MUC16 were quantified with western blotting, and expression of MUC1 and MUC16 mRNA was assessed with real-time PCR.

**Results:**

OSDI responses ranged from 0 to 60, and NITBUT ranged from 18.5 to 2.9 s. Only two statistically significant correlations were found: soluble MUC16 protein concentration and MUC16 mRNA expression with OSDI vision related (−0.47; p=0.01) and ocular symptom (0.39; p=0.02) subscores, respectively. Post hoc exploratory analysis on absolute expression values was performed on two subsets of subjects defined as asymptomatic (OSDI ≤6, n=12) and moderate to severe symptomatic (OSDI ≥20, n=12). The only significant difference between the two subgroups was a significant reduction in MUC16 mRNA expression found in the symptomatic dry eye group (1.52±1.19 versus 0.57±0.44; p=0.03).

**Conclusions:**

A broad exploration of mucin expression compared to either a sign (NITBUT) or symptoms of dry eye failed to reveal compelling evidence supporting a significant relationship, other than a potential association between MUC16 with specific symptoms. Furthermore, comparison of mucin protein and expression levels between the asymptomatic and moderate to severe symptomatic subgroups revealed only one significant difference, a reduction in MUC16 mRNA expression in the symptomatic subgroup.

## Introduction

Numerous compositional models of the tear film have been proposed. The first description by Wolff [1] in 1946 presented a three-layered tear film, consisting of an anterior lipid layer, a middle aqueous layer, and an inner mucin layer. As additional information became available, this model evolved to accommodate the possibility of soluble mucins in the aqueous layer, decreasing in concentration toward the lipid layer [2]. The present concept is that the tear film is a bilayered structure, consisting of an aqueous/mucinous phase and an outermost multilayered lipid phase [3].

Of the various components of the tear film, mucins are thought to play a key role in the retention of water and other tear fluid components on the ocular surface, facilitating a healthy, wet ocular surface. To date, at least 20 different mucin subtypes have been characterized [4-13], and of these, the secreted (mucin 2, cell surface associated [MUC2], MUC5AC, MUC5B, MUC7) and membrane-bound (MUC1, MUC4, MUC16) forms are expressed by ocular surface epithelia [14,15]. Of the mucins identified on the ocular surface, goblet cell–derived MUC5AC and three membrane-bound forms (MUC1, MUC4, MUC16) are the most relevant for maintaining a normal tear film [14]. Specifically, data support a role for mucins in such critical tasks as clearing debris and pathogens, protecting the corneal and conjunctival epithelium, preventing bacterial adhesion, and promoting boundary lubrication [16]. More recent evidence supports the fact that the ectodomain of each membrane-bound species is constitutively released into the tear film, forming soluble versions of these molecules. To date, the biologic role(s) associated with these soluble species have yet to be elucidated [17-21].

Alteration in mucin expression or mucin glycosylation has been implicated in the pathophysiology of dry eye. Reduction in the concentration of goblet cell–derived MUC5AC [22-24], membrane-bound or soluble forms of MUC1 and MUC16 [20,21,25-27], and conjunctival MUC1, MUC2, MUC4, and MUC5AC messenger ribonucleic acid (mRNA) [28] have been reported. Complicating the interpretation of these studies are various variables, including multiple study sub-populations (Sjögren’s syndrome, contact lens wearers, symptomatic young adults, keratoconjunctivitis sicca), varied dry eye inclusion criteria, multiple tear and conjunctival sample collection methods, and small sample sizes. Contradictory data also exist in the literature, where no change [20,21,25,29] or an increase in mucin mRNA or protein concentration [20,21] has been reported. Such apparent contradictions can seem more obvious when three different populations are compared simultaneously, as in the studies by Caffery et al. [20,21]. In these studies, patients with Sjögren’s syndrome routinely displayed higher amounts of soluble mucin and mRNA coding for MUC1 and MUC16 compared to the other two groups whereas generally no difference was found between mucin amounts when the non-Sjögren’s keratoconjunctivitis sicca (KCS) and control groups were compared. Last, despite the proposed importance of mucins in maintaining a healthy ocular surface and tear film, data supporting a correlation between mucin expression and signs or symptoms of dry eye are lacking [20,21,25,29]. Taken together, conclusions regarding the etiological or pathophysiological relevance between aberrant mucin expression and dry eye have been difficult to draw. Given the potential that a specific mucin mRNA or protein species could serve as a quantitative indicator of dry eye [28] or as a target for the therapeutic treatment of dry eye [30,31], additional studies seem prudent.

The results of large epidemiological studies [32] conducted in the United States clearly suggest that the prevalence of dry eye is greater in women than in men, and that women frequently consult clinicians with symptoms of ocular dryness and discomfort [33,34]. To date, only one large study has focused on postmenopausal women (PMW) complaining of dry eye disease [25]. In this study, PMW with a history of dry eye displayed significantly increased membrane-bound MUC1 and MUC16 protein and MUC1 mRNA levels compared to asymptomatic controls, prompting the hypothesis that increased mucin concentration may be a compensatory response to irritation.

In light of all the data presented above, the aim of this study was to further explore MUC1 and MUC16 protein and mRNA concentrations in a group of PMW. Given that signs and symptoms of dry eye are notoriously uncorrelated and little to no consistency exists in the literature regarding study design, we enrolled a cohort of PMW that would span a range of symptoms as well as one key sign of dry eye, tear stability. Tear stability was chosen based on the hypothesis that mucin significantly aids in aqueous adherence to the ocular surface, and thus, we rationalized that a reduction in tear film mucin concentration may be clinically characterized with alterations in the non-invasive tear break-up time (NITBUT).

## Methods

### Participants

This study received approval from the University of Waterloo’s Office of Research Ethics before initiation, and informed consent was obtained from all participants according to the tenets of the Declaration of Helsinki. A case history and complete ocular surface examination were performed to determine participant eligibility. Participants on hormone replacement therapy (HRT) were excluded, as were contact lens wearers and participants receiving any topical ocular medication or systemic medication known to exacerbate dry eye. Participants with a prior history of blepharitis or active blepharitis at the time of recruitment were also excluded from the study. “Postmenopausal” was defined as no menses for at least 1 year, not associated with hysterectomy. Thirty-nine healthy postmenopausal women (PMW) greater than 50 years of age were recruited (age range= 50-70 years).

### Subjective and objective clinical measurements

To assess symptoms, participants completed the Allergan Ocular Surface Disease Index (OSDI) questionnaire [35]. Tear stability was assessed by performing a NITBUT evaluation using the ALCON EyeMap (Model EH-290 Topography System, ALCON Inc., Fort Worth, TX). The keratoscope unit produces concentric rings of light, which are reflected off the cornea and imaged through a charge-coupled device (CCD) camera. NITBUT was quantified by measuring the time taken for distortions or discontinuities to appear in the reflected image of the concentric ring pattern. The time (in seconds) for the tear film to rupture (and thus distort the rings) was measured using a stopwatch, to the nearest 0.1 of a second. Three measurements were taken in each eye, and the overall average of both eyes was used for analysis.

### Analytical techniques

#### Reagents and materials

Agarose was from Cambrex Bio Science (Rockland, ME). Molecular weight standards (HiMark prestained protein standard) were from Invitrogen (Carlsbad, CA). ECL-Plus kits were from GE Healthcare (Baie d'Urfe, Canada). The 10X Complete protease inhibitor cocktail was from Roche (Mannheim, Germany). The DC Protein Assay Kit was from BioRad Laboratories (Mississauga, Canada). Mouse monoclonal antihuman MUC1 antibody (DF3) was from Signet (Dedham, MA), monoclonal mouse antihuman MUC16 antibody (OC125) was from DAKO (Glostrup, Denmark), and goat antimouse immunoglobulin G horseradish peroxidase was from Santa Cruz Biotechnology Inc. (Santa Cruz, CA). MUC16 standard antigen (CA125) and MUC1 standard antigen (CA15–3) were from Biodesign (Memphis, TN). The 0.45 μM pore Millipore Membrane Filters were from Millipore (Billerica, MA). The RNeasy Mini kit including RLT RNA Isolation buffer was from Qiagen Inc. (Mississauga, Canada). The SuperScript III First-Strand Synthesis System for real-time PCR (RT–PCR) was from Invitrogen (Carlsbad, CA), and the TaqMan Universal PCR Master Mix was from Applied Biosystems (Foster City, CA). DC Protein Assay kit was purchased from Biorad, Mississauga, Canada). All other chemicals were purchased from Sigma-Aldrich, (Oakville, Canada).

### Capillary tear collection

Using a graduated disposable 5 μl microcapillary tube (Wiretol-Micropipettes, Drummond Scientific Co., Broomall, PA), up to 5 μl of tears per eye were collected from the inferior temporal tear meniscus of each participant, without corneal anesthesia. Tears from both eyes were pooled and immediately placed on dry ice until transfer to −80 °C for storage.

### Conjunctival impression cytology

Following topical anesthesia (Alcaine, Alcon), epithelial cells were collected via impression cytology of the superior and temporal conjunctiva from each eye using sterile membrane filters cut to approximately 9 mm diameter circles. The two conjunctival impression cytology (CIC) samples from the right eye were placed in 1 ml of RLT RNA Isolation Buffer containing 0.01% β-mercaptoethanol, whereas the two left eye samples were placed in an empty sterile 2 ml tube, thus facilitating the isolation of total RNA and total protein, respectively. All samples were immediately placed on dry ice and then transferred to −80 °C for storage until processing.

### Protein isolation from conjunctival impression cytology samples

Total protein was isolated using 75 µl of extraction buffer (2% w/v sodium dodecyl sulfate [SDS]), 1X Complete protease inhibitor cocktail in 50 mM Tris-HCL buffer pH 7.4 (Tris: 2-Amino-2-hydroxymethyl-propane-1,3-diol). Following vigorous mixing, the samples were heated at 95 °C for 10 min. Tubes were centrifuged at 12 000 × *g* (RCF) for 6 min. Then the protein extract was collected, transferred to a fresh, capped polypropylene centrifuge tube, and frozen at −80 °C until analysis.

### Determination of total protein concentration in tear and conjunctival impression cytology samples

All total protein determinations were conducted using the DC Protein Assay Kit, following the manufacturer’s instructions. Five µl of each CIC protein extract and 1 µl of each tear sample were diluted to a total of 10 µl in distilled water and assayed in duplicate.

### Electrophoresis and immunoblotting

Samples were thawed at room temperature and diluted to the appropriate final concentrations in sample buffer (247 mM Tris-HCl, pH 8.6, 2% SDS (w/v), 50 mM dithiothreitol, 1X Complete Protease Inhibitor, 10% glycerol, 0.002% (w/v) Bromophenol blue). Protein samples were subjected to agarose gel electrophoresis using a SE600 vertical gel unit (Hoefer Scientfic, San Francisco, CA). A titration of MUC16 standard antigen (CA125) or MUC1 standard antigen (CA15–3) was run on each gel to normalize data and facilitate semiquantitation of the samples, through linear regression analysis. For MUC1, 6 µg of total tear protein or 20 µg of CIC total protein extract was loaded per lane (determined from preliminary experiments, data not shown). For MUC16, 4.0 µg of tear total protein or 5.0 µg of total CIC protein was loaded per lane. Following separation, the protein was vacuum transferred to nitrocellulose membranes. Membranes were fixed by heating at 70 °C for 30 min, air dried for 12 h, and then blocked 0.1% w/v bovine serum albumin (BSA) in phosphate buffered saline (PBS; 137 mM sodium chloride, 2.7 mM potassium chloride, 10 mM sodium phosphate, pH 7.4) + 0.05% v/v Tween-20 (=PBS-T) for 1 h at room temperature. Blots were incubated overnight in appropriate antibody (DF3, 1: 40 or OC 125, 1:250) diluted in PBS-T and 0.1% BSA at 4 °C. After rinsing, blots were incubated with secondary antibody (1:5000) in PBS-T + 0.1% BSA for 1 h at room temperature. Blots were developed with ECL Plus and chemiluminescent signals were imaged with a Storm 840 Workstation (Molecular Dynamics; GE Healthcare Life Sciences, Baie d'Urfe, Canada). The amount of MUC1 or MUC16 in each sample and standard were quantified with image analysis software (ImageQuant 5.1, Molecular Dynamics). Known amounts of CA15–3 or CA125 were used to generate standard curves, and using the line-of-best-fit from the standard curve, the relative amount of MUC1 or MUC16 was interpolated from the graph. All samples produced multiple chemiluminescent signals of varying molecular weights. For quantitation, only signals equal to and above 150 kDa were used. All data are described in units of MUC 1 or MUC 16 standard per microgram total protein.

### Ribonucleic acid isolation and reverse transcription

Tubes containing 1 ml of RLT buffer and two impression cytology samples were allowed to thaw at room temperature and then vortexed for 30 s. Membranes were removed, and samples were passed through a 21 gauge needle ten times. Extraction of total RNA proceeded according to the manufacturer’s instructions using the DNase step as recommended. The final isolation step was conducted with 40 µL of RNase free water. cDNA was synthesized from 8 µl of the RNA sample using random hexamer primers with the SuperScript III First-Strand Synthesis System for RT–PCR according to the manufacturer’s instructions.

### Real-time quantitative polymerase chain reaction

Multiplex PCR reactions containing target (*MUC1* or *MUC16*) and endogenous control (glyceraldehyde 3-phosphate dehydrogenase) oligonucleotide primers were performed in the presence of gene-specific dye-labeled TaqMan probes (Table 1). Briefly, 2 μl of cDNA was used for amplification in a 50 µl PCR reaction containing the target and endogenous control oligonucleotide primers, control and target TaqMan probes, and TaqMan Universal PCR Master Mix. Duplicate samples were used for analysis in a 7500 Real-Time PCR System (Applied Biosystems). Conditions used for amplification were as follows: 50 °C for 2 min and then an initial 10 min denaturing step at 95 °C. This was followed by 40 cycles of denaturing at 95 °C for 30 s, annealing at 60 °C for 30 s, and extension at 72 °C for 45 s. Normalized reporter dye fluorescence (R_n_) data were collected during the extension step at each cycle. The collected data were analyzed, and fold-expression changes were calculated using the comparative method (2^-ΔΔCT^) of relative quantification with SDS software (v1.3.1; Applied Biosystems). Final data are expressed in relative quantification (RQ) units.

**Table 1:** Sequence of Primers and Probes Used for Gene Amplification in Real Time RT–PCR

**Gene**	**Forward primer**	**Reverse primer**	**Taqman probe**
MUC1	CTGGTCTGTGTTCTGGTTGC	CCACTGCTGGGTTTGTGTAA	6FAM-GAAAGAACTACGGGCAGCTG
MUC16	ACCCAGCTGCAGAACTTCA	GGTAGTAGCCTGGGCACTGT	6FAM-GCGGAAGAAGGAAGGAGAAT
GAPDH	GAAGGTGAAGGTCGGAGTCA	GACAAGCTTCCCGTTCTGAG	VIC-CAATGACCCCTTCATTGACC

### Statistical analysis

Statistical analysis was performed using Statistica Ver7.1 (StatSoft Inc., Tulsa, OK) and Microsoft Excel XLfit software (Microsoft, Mississauga, Canada). Graphs were plotted using Excel. All data are reported as mean±standard deviation. The relationship between mucin expression and either OSDI or NITBUT data was calculated via Pearson linear correlation (StatPlus:mac LE2009). Statistical differences between asymptomatic and symptomatic groups were identified with the Student *t* test comparison of means. Significance was identified at p<0.05 (α=0.05).

## Results

In this study, the presence and severity of dry eye were assessed by determining symptoms and tear stability (Table 2). Following our clinical study design, clinical data were used only for correlation purposes and did not define inclusion. Post hoc analysis addressed whether relative extremes in symptoms defined as asymptomatic (OSDI score=0–6) and symptomatic (OSDI score ≥20) were associated with differential expression of mucin. A summary of all clinical data is presented in Table 2 and Table 3. No correlation was found between NITBUT and OSDI, nor was a significant difference found between OSDI and NITBUT when compared based on the presence or absence of moderate symptoms (Table 3).

**Table 2:** Summary of Clinical Data Associated with Complete Cohort of PMW Enrolled in Study (n=39)

**Measure**	**Value**	**Range**
Mean Age	61.4±8.5	52–79
Mean Total OSDI Score	17.7±15.3	0–60
Mean Ocular Symptoms OSDI SubScore	16.8±16.4	0–65
Mean Vision Related Function OSDI SubScore	16.1±15.1	0–56
Mean Environmental OSDI SubScore	21.4±25.8	0–92
Mean NITBUT	5.31±2.8 s	2.9–18.5 s

**Table 3:** Summary of Clinical Data Associated with Sub Group Analysis

**Measure**	**Asymptomatic** **(n=12)**	**Symptomatic** **(n=12)**	**p**
Mean Age	62.4±9.9	58.4±4.6	0.22
Mean Total OSDI Score	2.3±2.2	35.2±12.1	<0.0001*
Mean Ocular Symptoms OSDI SubScore	4.2±4.7	34.2±16.8	<0.0001*
Mean Vision Related Function OSDI SubScore	1.0±2.4	30.7±12.3	<0.0001*
Mean Environmental OSDI SubScore	0.7±2.4	43.1±27.7	<0.0001*
Mean NITBUT	6.7±4.4 s	4.7±1.6 s	0.15

* denotes statistical significance (p<0.05)

### Mucin quantitation

**Figure 1:**
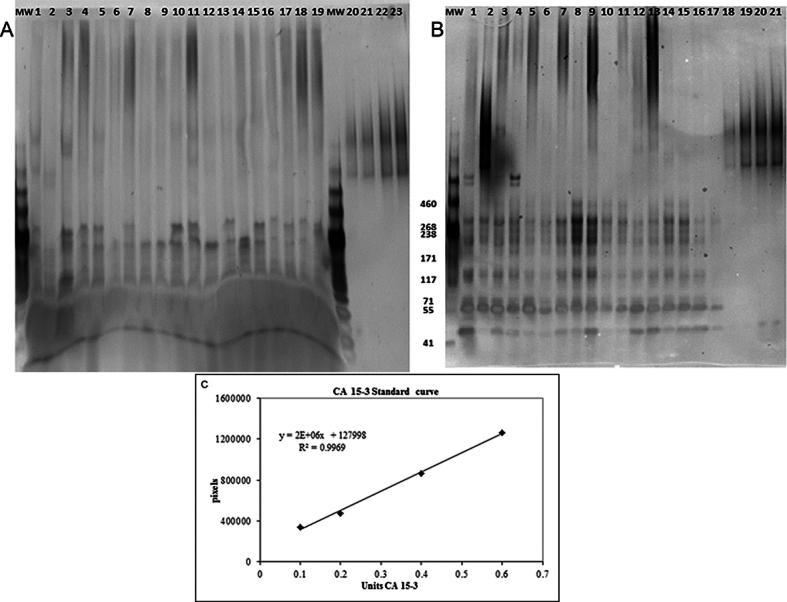
Western blot. Panel **A** s an example of MUC1 western blot from impression cytology samples (membrane bound mucin 1 [MUC1]). Lanes 1–19 are participant samples. Lanes 20–23 are MUC1 standards (CA15–3; 0.1, 0.2, 0.4, and 0.6 units). MW are molecular weight markers from 41 to 460 kDa (kD) beside the accompanying band. Panel **B** is an example of MUC1 western blot from tear samples (soluble MUC1). Lanes 1–17 are participant samples. Lanes 18–21 are MUC1 standard (CA15–3; 0.1, 0.4, 0.8, and 1 unit). **C**: The sample regression curve (from **A**) was created by graphing applied concentration of MUC1 standard (CA15–3) against the optical density of the resulting band immunoreactivity. Total MUC1 concentration was quantified by interpolation from this curve. For analysis purposes, all sample chemiluminescent signals at and above 150 kDa were used.

**Figure 2:**
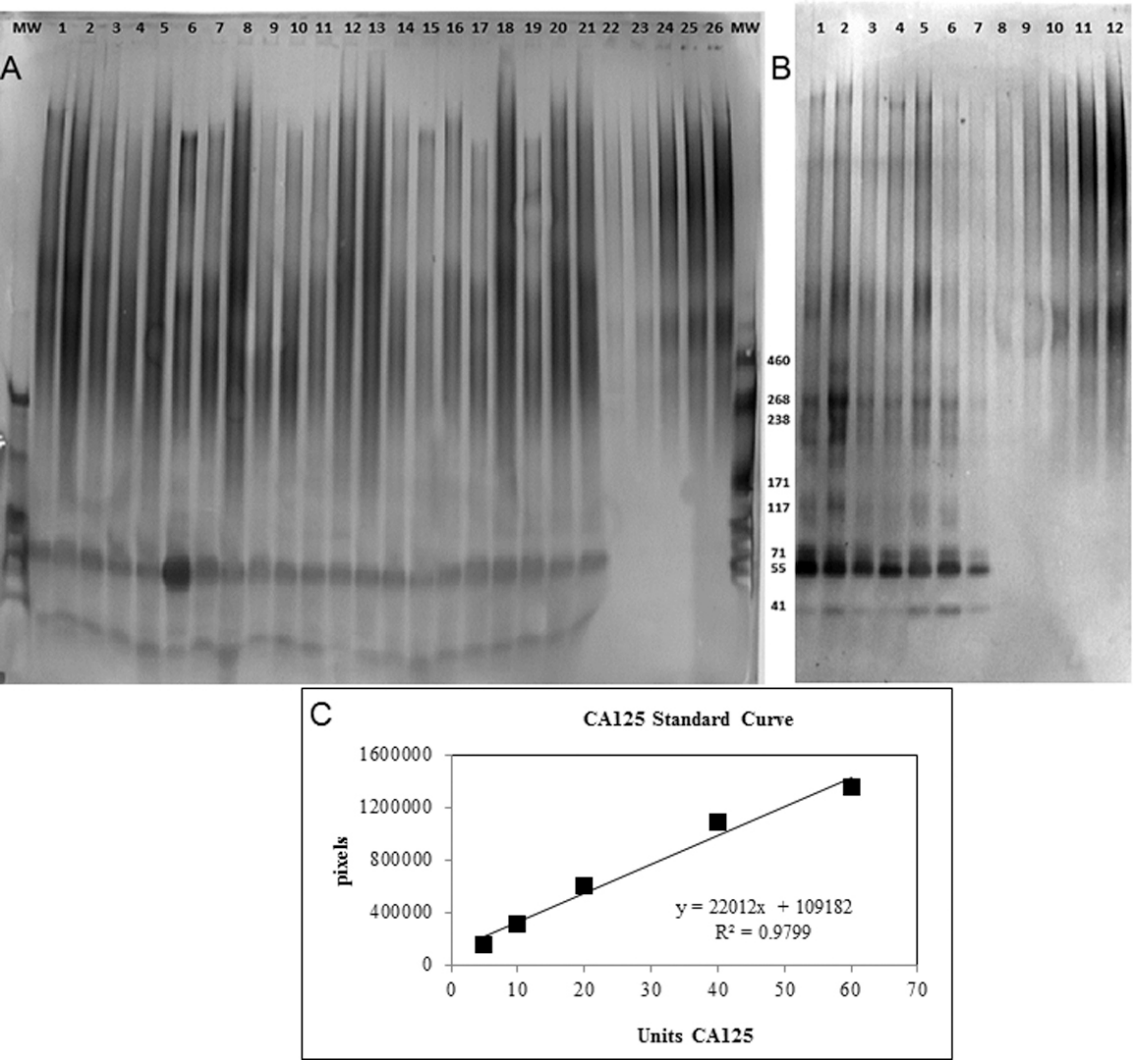
Western blot. Panel **A** is an example of MUC16 western blot from impression cytology samples (membrane bound MUC16). Lanes 1–21 are participant samples. Lanes 22–26 are MUC16 standards (CA125; 5, 10, 32, 60, and 74 units). MW are molecular weight markers from 41 to 460 kDa (kD) beside the accompanying band. Panel **B** is an example of MUC16 western blot from tear samples (soluble MUC16). Lanes 1–7 are participant samples. Lanes 8–12 are MUC16 standard (CA125; 5, 10, 20, 40, and 60 units). **C**: The sample regression curve (from **B**) was created by graphing the applied concentration of MUC16 standard (CA125) against the optical density of the resulting band immunoreactivity. Total MUC16 concentration was quantified by interpolation from this curve. For analysis purposes, all sample chemiluminescent signals at and above 150 kDa were used.

In some cases, there was insufficient sample material to perform all the mucin assays; thus, the number of data points per analysis varies, as noted in Table 4, Table 5, and Table 6. Figure 1 and Figure 2 display representative quantitative data describing mucin protein concentration.

**Table 4:** Summary of Correlations Between NITBUT and Mucin Expression

**MUC of Interest**	**Pearson Correlation Coefficient (NITBUT versus MUC of Interest)**	**p value**	**n**
Tear Film MUC1	−0.29	0.08	37
Tear Film MUC16	0.05	0.79	26
Membrane Bound MUC1	−0.16	0.35	36
Membrane Bound MUC16	−0.12	0.48	36
MUC1 mRNA	−0.12	0.50	33
MUC16 mRNA	−0.31	0.08	33

**Table 5:** Summary of Correlations Between Total OSDI Score and OSDI Sub Scores with Mucin Expression

**MUC of Interest**	**Pearson Correlation Coefficient** **[p value]**	**Pearson Correlation Coefficient** **[p value]**	**Pearson Correlation Coefficient [p value]**	**Pearson Correlation Coefficient [p value]**
	***TOTAL OSDI SCORE***	***OCULAR SYMPTOM SUBSCORE***	***VISON*** ***RELATED*** ***SUBSCORE***	***ENVIRON SUBSCORE***
Tear Film MUC 1	−0.11 [0.51]	−0.06 [0.70]	−0.10 [0.57]	−0.12 [0.47]
Tear Film MUC16	−0.33 [0.10]	−0.18 [0.39]	−0.47 [0.01]*	−0.25 [0.22]
Membrane Bound MUC1	0.02 [0.89]	−0.03 [0.86]	0.03 [0.88]	0.07 [0.68]
Membrane Bound MUC16	0.05 [0.77]	0.18 [0.29]	−0.02 [0.92]	−0.06 [0.72]
MUC1 mRNA	0.07 [ 0.69]	0.12 [0.50]	−0.03 [0.88]	0.05 [0.76]
MUC16 mRNA	0.33 [0.06]	0.39 [0.02]*	0.12 [0.51]	0.27 [0.13]

* denotes statistical significance (p<0.05).

**Table 6:** Summary of Mucin Protein and mRNA Expression Data

**MUCIN PROTEIN EXPRESSION DATA: Data Expressed in units per microgram total protein**				
Tear Film MUC1	Capillary tears	**Asymptomatic**	**Symptomatic**	**p value**
		0.04±0.09 (n=11)	0.03±0.03 (n=12)	0.6
Tear Film MUC16	Capillary tears	2.73±1.30 (n=10)	2.20±1.88 (n=8)	0.51

Membrane Bound MUC1	CIC	0.012±0.013 (n=12)	0.014±0.012 (n=10)	0.75
Membrane Bound MUC16	CIC	14.91±9.48 (n=10)	16.63±8.45 (n=10)	0.70
**MUCIN RNA EXPRESSION DATA: Data Expressed in Mean RQ Units**				
MUC1 mRNA	CIC	0.82±0.40 (n=11)	0.93±0.30 (n=9)	0.49
MUC16 mRNA	CIC	0.57±0.44 (n=11)	1.52±1.19 (n=9)	0.03*

* denotes statistical significance (p<0.05)

### Correlation between mucin expression, the Ocular Surface Disease Index, and non-invasive tear breakup time

Table 4 and Table 5 summarize results from all correlation analyses, which revealed weak and insignificant correlations for all comparisons (NITBUT, total OSDI score, and individual OSDI subscores), with the exception of MUC16 mRNA expression and soluble MUC16 protein concentration with OSDI vision related and ocular symptom subscores, respectively.

### Mucin 1, cell surface associated and mucin 16, cell surface associated proteins and messenger ribonucleic acid in tears and conjunctival epithelial cells derived from asymptomatic and symptomatic participants: A post hoc subgroup analysis

As presented in Table 6, direct comparison of a subset of data from this study indicated that soluble and membrane-bound MUC1 and MUC16 concentration remained invariant between the symptomatic and asymptomatic study groups. The same comparison also revealed that MUC1 mRNA expression remained unchanged, whereas significant downregulation (p=0.03) of MUC16 mRNA was found in the symptomatic group.

## Discussion

In this study, we explored the relationship between the quantity of MUC1 and MUC16 protein and mRNA with dry eye symptoms and tear stability in a cohort of PMW that were recruited without bias of specific inclusion criteria. We chose this objective and clinical design because significant confusion exists in the literature about the potential role altered mucin expression may play in dry eye. Specifically, data exist supporting increased, decreased, and no change in mucin concentration associated with dry eye. Complicating interpretation of these results is a multitude of study variables, including different dry eye subgroups, inclusion and exclusion criteria, study population size, and analytical methods. Thus, beyond our group’s research interests in PMW, stepping back and examining the broader picture of whether any significant findings could be uncovered was of value. Based on our analysis, including a post hoc exploration of absolute mucin concentration in asymptomatic control and moderate symptomatic dry eye subjects, we conclude that, at least in PMW, mucin expression has no obvious role in either dry eye symptomology or the maintenance of tear stability.

Data from this study generally failed to uncover any correlation between MUC1 or MUC16 protein concentration or MUC1 mRNA expression compared to a range of symptom data (total OSDI scores ranging from 0 to 60 and all associated sub scores) or tear stability data (range, 18.5–2.9 s). The two significant, albeit marginally, correlated findings were MUC16 mRNA expression and soluble MUC16 protein concentration with OSDI ocular symptom and vision related subscores, respectively.

Following the lack of compelling findings in our correlation survey, a subgroup analysis was performed post hoc to separate out those subjects who were essentially void of dry eye symptoms (OSDI ≤6) compared to those with moderate to severe symptoms (OSDI ≥20) to examine the hypothesis that a biologic signal could be discerned only with more moderate disease. This exploration also failed to uncover significant findings, with the exception of downregulation in MUC16 mRNA expression in the symptomatic group.

Our data are in partial agreement with a recently published study [25], which concluded that symptomatic PMW appear to launch a compensatory response to ocular irritation through significant upregulation in mucin expression. In general, our data failed to demonstrate significant correlations, and where significance was found, we concluded that the increasing symptoms were associated with increased expression of MUC16 mRNA but lower tear film MUC16. Both studies used symptoms as a focal point, with Gipson et al. [25] using symptoms as the primary means of defining dry eye and our study performing a subgroup analysis to explore this association specifically. Different questionnaires were used; thus, gauging the severity of symptoms in the two studies is difficult, leaving open the possibility that mucin expression is sensitive to small changes in symptoms, although this is unlikely. In Gipson et al.’s study, additional inclusion criteria were used, including documented history of dry eye ≥3 months and current use of artificial tears. Thus, a potentially more advanced form of dry eye may have been sampled in Gipson et al.’s study, as in our subgroup analysis, no significant difference in tear stability was found between the two groups. Perhaps of greatest note is study size. Although our study initially enrolled 39 subjects, which is larger than a typical “biomarker” study, it is clear from the data standard deviations that much larger sample sizes (such as that employed by Gipson et al.) may be needed to gain insight into true data significance.

Our findings are largely in contrast with the majority of published data, which generally support the hypothesis that altered (up- or downregulation) mucin expression is associated with dry eye. However, several consistent findings were found, including reports by Argüeso et al. [22] and Caffery et al. [20,21], who reported no or few differences in the expression of MUC1, MUC4, or MUC16 mRNA or protein expression between keratoconjunctivitis sicca (KCS) and control groups.

In conclusion, our study of 39 PMW failed to uncover a significant association between mucin expression and either dry eye symptoms or tear stability. Furthermore, in a post hoc exploratory review of a small subset of our data polarizing groups between asymptomatic and moderate symptomatic, the only difference in mucin expression differentiating the groups was a reduction in MUC16 mRNA. Given the overall lack of consistency within the mucin expression literature as a whole in addition to the lack of evidence supporting a clinical correlation between mucin expression and presence or absence of dry eye, concluding what, if any role(s) alteration in mucin expression plays in the pathophysiology of dry eye or the validity of targeting mucin expression as a therapeutic treatment strategy for dry eye is difficult.
